# Seasonal Variation in the Responsiveness of the Melanopsin System to Evening Light: Why We Should Report Season When Collecting Data in Human Sleep and Circadian Studies

**DOI:** 10.3390/clockssleep5040044

**Published:** 2023-11-01

**Authors:** Isabel Schöllhorn, Oliver Stefani, Christine Blume, Christian Cajochen

**Affiliations:** 1 Centre for Chronobiology, Psychiatric Hospital of the University of Basel, 4002 Basel, Switzerland; isabel.schoellhorn@unibas.ch (I.S.); oliver.stefani@hslu.ch (O.S.);; 2 Research Cluster Molecular and Cognitive Neurosciences (MCN), University of Basel, 4001 Basel, Switzerland; 3 Lucerne University of Applied Sciences and Arts, Engineering and Architecture, Technikumstrasse 21, 6048 Horw, Switzerland; 4 Department of Biomedicine, University of Basel, 4001 Basel, Switzerland

**Keywords:** season, light sensitivity, non-image forming effects, melatonin, sleep-latency

## Abstract

It is well known that variations in light exposure during the day affect light sensitivity in the evening. More daylight reduces sensitivity, and less daylight increases it. On average days, we spend less time outdoors in winter and receive far less light than in summer. Therefore, it could be relevant when collecting research data on the non-image forming (NIF) effects of light on circadian rhythms and sleep. In fact, studies conducted only in winter may result in more pronounced NIF effects than in summer. Here, we systematically collected information on the extent to which studies on the NIF effects of evening light include information on season and/or light history. We found that more studies were conducted in winter than in summer and that reporting when a study was conducted or measuring individual light history is not currently a standard in sleep and circadian research. In addition, we sought to evaluate seasonal variations in a previously published dataset of 72 participants investigating circadian and sleep effects of evening light exposure in a laboratory protocol where daytime light history was not controlled. In this study, we selectively modulated melanopic irradiance at four different light levels (<90 lx). Here, we aimed to retrospectively evaluate seasonal variations in the responsiveness of the melanopsin system by combining all data sets in an exploratory manner. Our analyses suggest that light sensitivity is indeed reduced in summer compared to winter. Thus, to increase the reproducibility of NIF effects on sleep and circadian measures, we recommend an assessment of the light history and encourage standardization of reporting guidelines on the seasonal distribution of measurements.

## 1. Introduction

Light is the most important zeitgeber for the internal biological clock in the brain, located within the suprachiasmatic nuclei (SCN) [1]. To allow for synchronization with the environment, the SCN has direct connections to the intrinsically photosensitive retinal ganglion cells (ipRGCs) in the retina, which contain the photopigment melanopsin and are most sensitive to short-wavelength light (~480 nm) [2,3,4]. The photic information received by the SCN is then relayed to the pineal gland, resulting in a circadian pattern of serotonin and melatonin production. Circadian melatonin rhythms [5], sleep timing [5], and sleep architecture [6] show seasonal variations. Moreover, Münch et al. [7] and Kawasaki et al. [8] found seasonal variations in the post-illumination pupil response, which is known to be regulated by the melanopsin system. In this publication, we aim to observe seasonal variations in the responsiveness of the melanopsin system by using light conditions that were matched for S-, M- and L-cone excitation but differed in melanopic irradiance (high vs. low melanopic irradiance; Method of Silent Substitution [9]). Because melanopic irradiance predicts melatonin suppression [10,11,12] and sleep latency [12], these variables were used for the analysis of seasonal dependencies. In addition to their role as a pacemaker for circadian rhythms and their photic entrainment with the environmental light-dark cycle, the SCN also helps to regulate seasonal rhythms [13]. A stronger circadian entrainment in summer than in winter [5] has been attributed to increased motor activity levels and daylight exposure when the photoperiod becomes longer [14]. Therefore, less light during winter days may lead to an increased sensitivity to evening light. Several studies have indeed shown that reduced light levels prior to evening light exposure increase light sensitivity as indexed by more melatonin suppression [15,16,17,18] and a stronger alerting response [18]. Therefore, a study on non-image forming (NIF) effects of light may yield different results in summer and winter, especially if light history is not controlled. As part of a systematic review, we collected information on the seasonal distribution and light history from relevant publications. Our aim is to provide a broader picture of the seasonal distribution of studies on the effects of evening light on melatonin and sleep and to derive recommendations on how to improve the reproducibility of studies in the field of circadian rhythm and sleep research.

Here, we (1) assessed whether seasonal and light history information is available in the literature on the effects of evening light exposure on melatonin secretion and polysomnographically assessed sleep. In addition, (2) the effects of melanopic irradiance on melatonin and sleep latency (published in [12]) were analyzed according to season. To this end, we used a data set of 72 young, healthy male participants who were exposed to relatively low light levels (<90 lx) of screen light that differed in melanopic irradiance (high- vs. low-melanopic; HM vs. LM) but were matched in terms of luminance (27 cd/m^2^, 62 cd/m^2^, 135 cd/m^2^, 284 cd/m^2^) and for S, M, and L cone excitation.

## 2. Results

### 2.1. Reporting of Season in Sleep and Circadian Research on Evening Light Effects

#### 2.1.1. Evening Melatonin Concentrations

**Figure 1:**
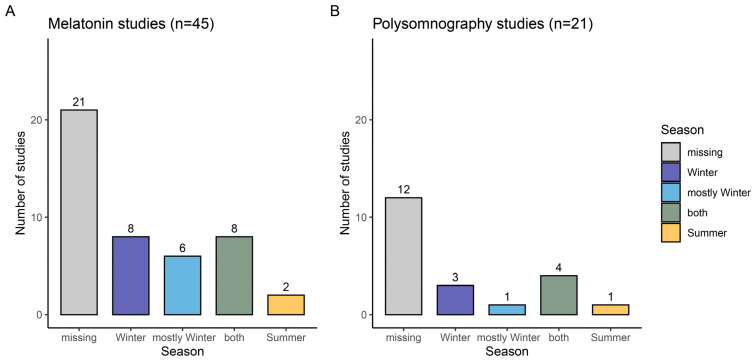
Reporting of season or period of data collection: (**A**) Studies on evening melatonin concentration; (**B**) Studies assessing sleep with polysomnography following evening light exposure. The grey bars correspond to the number of manuscripts that did not include information about the season and period of data collection. The darker blue bars refer to studies that collected data between October and March. The lighter blue bars represent the studies that were mostly conducted in winter (i.e., >3 months in winter and September or April). The green bars correspond to studies conducted in summer and winter. The studies in which the data collection took place in summer (i.e., between April and September) are shown in yellow.

In this systematic review, we only included studies that investigated the impact of evening light exposure (i.e., between 5:30 p.m. and 2 a.m.) on melatonin secretion. Studies with light interventions during daytime or at night were not included. We extracted data from 45 laboratory studies selected according to our inclusion criteria (see Methods section, [12,19,20,21,22,23,24,25,26,27,28,29,30,31,32,33,34,35,36,37,38,39,40,41,42,43,44,45,46,47,48,49,50,51,52,53,54,55,56,57,58,59,60,61,62]). The season or time period (e.g., months) of data collection was explicitly reported in 53% of the studies (Figure 1A). Only two studies were solely conducted in summer. In more than 50% of the studies in which a time period was specified, data acquisition took place mainly in the winter (i.e., between October and March). Only a small proportion of studies kept participants in the laboratory throughout the experimental day to control for light history (*n* = 4) or recorded light history (*n* = 4). Light history was assessed subjectively (*n* = 1, [12]) or objectively using sensors integrated into actigraphs (*n* = 3, [21,27,41]) or spectacle frames (*n* = 1, [60]). The extracted information on season and light history can be found in the Supplementary Material.

#### 2.1.2. Polysomnographically Assessed Night-Time Sleep

In total, we extracted data from 21 studies that met our inclusion criteria [12,20,21,43,45,63,64,65,66,67,68,69,70,71,72,73,74,75,76,77,78]. Five of the studies have already been included in the results for melatonin. Only 43% of the studies (*n* = 9) specified the study period or season (Figure 1B). Of these, four studies were conducted during winter or mostly during winter, four studies during summer and winter and one study during summer. Nineteen percent (*n* = 4) of the studies kept participants in the laboratory under reduced light levels for the entire experimental day prior to sleep assessment, and only one study objectively recorded the light history [21]. The extracted seasonal and light history information can be found in the Supplementary Material.

### 2.2. Seasonal Sensitivity to Evening Light without Controlling Light History

Here, we report the results of a re-analysis investigating seasonal variations in the effects of evening light on melatonin and sleep latency. Therefore, we combined the datasets of all four luminance levels and conducted separate analyses for data collection during summer (April–September) and Winter (October–March).

#### 2.2.1. Solar Irradiance and Self-Reported Light History

**Figure 2:**
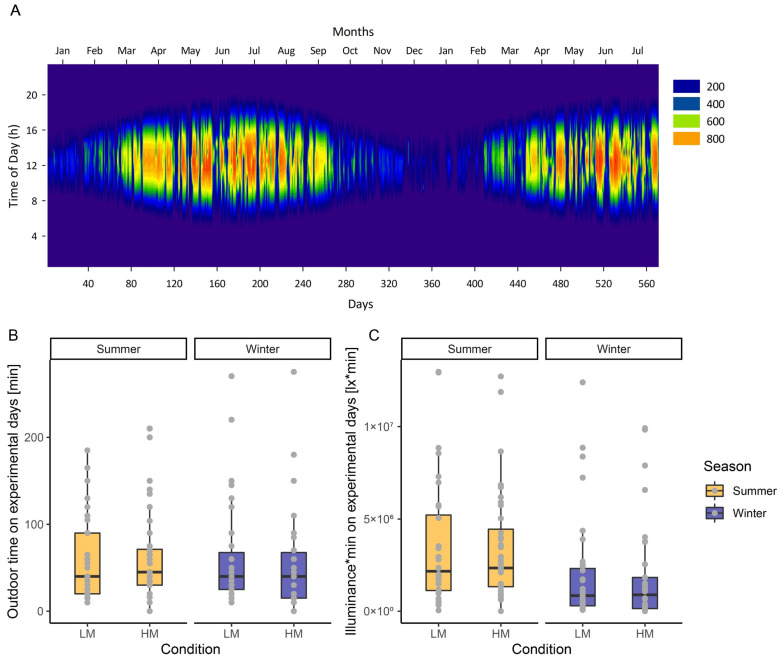
(**A**) Solar irradiance (W/m^2^) during the study period depending on the time of day measured in Basel (Klingelbergstrasse 47.561697, 7.580502, 264.00 m). (**B**) Time spent outdoors prior to the arrival at the laboratory on experimental days in summer and winter for each light condition. (**C**) Approximation of illuminance*min using reported exposure times and durations and solar irradiance (W/m^2^). To estimate the lux*minutes, we approximated 120 lx per 1 W/m^2^ (according to [79]), multiplied it by the minutes spent outdoors per hour of day and summed it up for each experimental day. The colored boxes indicate whether the data was collected in summer (yellow) or winter (purple). The lower and upper hinges of each box correspond to the first and third quartiles (the 25th and 75th percentiles). The black horizontal bar within each box refers to the median. The upper (lower) whisker extends from the hinge to the largest (lowest) value no further than 1.5 * the inter-quartile range (IQR). The individual values are highlighted in grey.

Participants arrived at the laboratory 7 h before their usual bedtime. Before entering the laboratory, participants were not restricted in their exposure to light, and neither did we objectively measure light exposure during the preceding day. However, solar irradiance for the study location, Basel (47.56°, 7.58°), was available for the study period (Figure 2A), and we asked participants how long and when they had spent time outdoors. Figure 2B illustrates the time spent outdoors in the morning, in the afternoon and throughout the day before arriving at the laboratory according to light conditions and season. There was no significant difference in the reported time spent outdoors on the experimental days between the two light conditions (HM vs. LM) nor between the different seasons. However, using solar irradiance and reported time spent outdoors as an approximation of lux*min, there was a significantly higher light exposure during the summer months compared to the winter months (F_1,78_ = 8.22, *p* < 0.01, ω^2^ = 0.08).

#### 2.2.2. Seasonal Dependent Light Sensitivity

**Figure 3:**
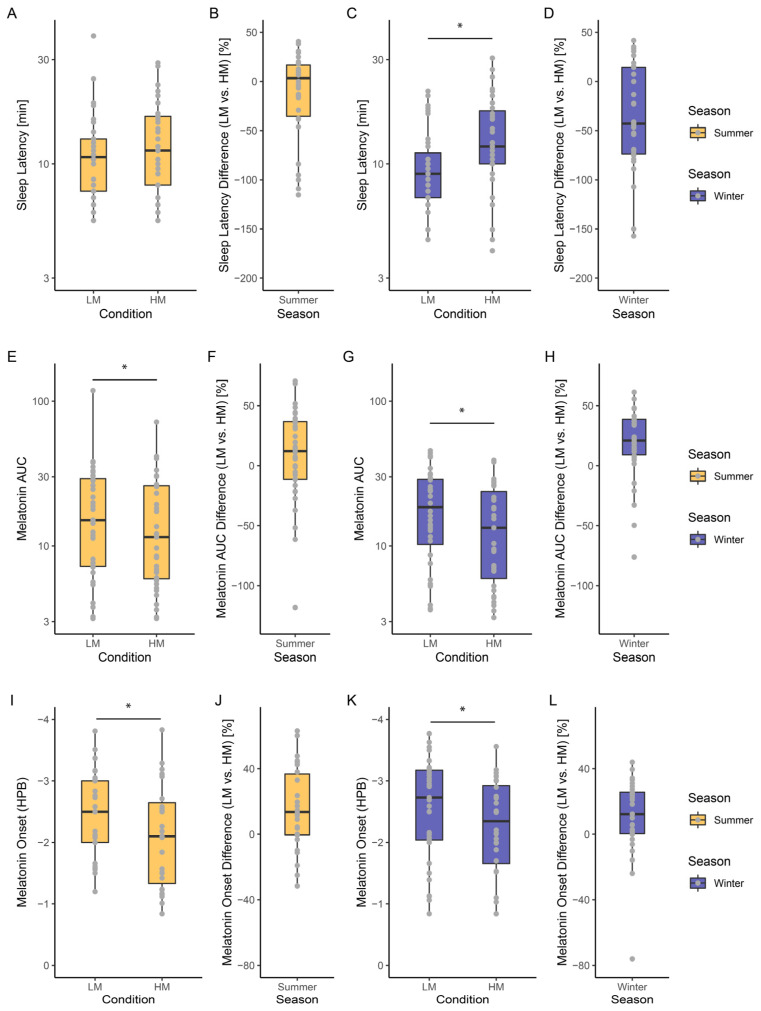
Boxplots of light effects depending on the season: (**A**–**D**) Sleep Latency in minutes on a log scale; (**E**–**H**) Melatonin Area Under the Curve (AUC); (**I**–**L**) Melatonin Onset in hours prior to bedtime (HPB). The difference [%] corresponds to (LM − HM)/LM. The coloured boxes indicate whether the data was collected in summer (yellow) or winter (purple). The lower and upper hinges of each box correspond to the first and third quartiles (the 25th and 75th percentiles). The black horizontal bar within each box refers to the median. The upper (lower) whisker extends from the hinge to the largest (lowest) value no further than 1.5 * the inter-quartile range (IQR). The individual values are highlighted in grey. Supplementary Figure S2 shows the light effects depending on the season for the four light intensity groups. Asterisks indicate a significant difference between the light conditions. Abbreviations: HPB—Hours prior to bedtime, LM—Low Melanopic Condition; HM—High Melanopic Condition.

During summer, there was no significant light-dependent effect on sleep latency (Light Condition: F_1,33_ = 1.40, *p* = 0.25) (Figure 3A,B). In contrast, during the winter months, there was a significant prolongation in sleep latency following the high compared to the low melanopic condition (Light Condition: F_1,34_ = 13.80, *p* < 0.001, ω^2^ = 0.26) (Figure 3C,D).

Effects of light exposure on the Area Under the Curve (AUC) of melatonin during summer (Light Condition: F_1,35_ = 4.25, *p* < 0.05, ω^2^ = 0.08) and winter (Light Condition: F_1,33_ = 15.41, *p* < 0.001, ω^2^ = 0.29) were significant (Figure 3E–H). However, the effects were more pronounced during the winter season, as indicated by the effect size.

The Melatonin Onset during light exposure in summer (Light Condition: F_1,27_ = 8.58, *p* = 0.007, ω^2^ = 0.21) and winter (Light Condition: F_1,29_ = 11.97, *p* = 0.002, ω^2^ = 0.26) were significant (Figure 3I–L).

## 3. Discussion

Laboratory studies investigating the effects of evening light exposure on melatonin secretion or PSG-assessed sleep are extremely time- and resource-consuming. To reduce the complexity of the experiment, researchers often refrain from assessing light exposure prior to the arrival at the lab. Additionally, it is often not feasible to control for seasonal effects as scheduling participants for protocols often comprising several weeks is already complex enough. Beyond this, climate change may make it challenging to run studies during the summer months if air conditioning is not available. Last, researchers often argue that acquiring data uniformly across seasons would rule out an effect of the season in their data. While this may be true, this makes it difficult to replicate and compare results, especially if information on when data were acquired is missing and/or individual light histories are not assessed. To circumvent such problems, we suggest here a hierarchy of measures, the most simple of which should be reported in any study investigating the effects of light on the biological clock and sleep.

In line with the assumptions outlined above, our systematic review yielded that the time period or season in which data collection took place was often not specified (i.e., only about 50% specified this). However, from the studies that did report the data collection period, more studies were conducted in the winter months than in the summer months. This is problematic because it is difficult to extrapolate the light effects quantified in winter to summer. The reason is that daylight exposure is significantly higher in summer than in winter, which in turn affects the sensitivity to evening light exposure [80,81,82]. In a study by Adamsson and colleagues [81], light radiation in summer, with a daily mean of 1,394,200 lux*min, was about 15 times higher than in winter (daily mean = 91,366 lux*min) and also higher than in spring (daily mean = 356,367 lux*min) and autumn (daily mean = 477,165 lux*min) at a northern latitude of 56° N, with large annual variations in photoperiod length. This is in line with the light levels (measured by wrist-worn actimeters) in the study by Zerbini et al., which were 10 times higher (on average) in summer than in winter assessed in Groningen (53°13′ N/6°33′ E) [5].

### 3.1. Seasonal Variations in Humans

Although the amplitude of seasonal variation is likely to be smaller in humans than in other animals, several physiological parameters have been suggested to still follow a seasonal pattern. For instance, caloric intake, and especially carbohydrate intake, has been found to be higher in autumn [83]. Blood pressure [84] and cholesterol levels [85] have also been reported to be higher in winter than in summer. Behavioral patterns also show seasonal variations, at least in pre-industrial societies, where, for example, conception peaked in spring and winter [86]. Variations in mood are probably the best-studied seasonal pattern, with the prevalence of major depressive disorder [87] and subclinical mood deterioration [88] increasing during the winter months. Serotonin may play a key role in seasonal mood changes and seasonal affective disorder; its production in the brain has been shown to be directly affected by the duration of sunlight [89]. In addition, a recent study by Meyer and colleagues [90] suggests that there are seasonal variations in task-related brain responses.

### 3.2. Seasonal Variation in Light-Induced Melatonin Suppression

Several studies have shown that reduced light levels during daytime increase nocturnal light sensitivity as assessed by melatonin suppression [15,16,17]. Thus, the effects of light may well be specific to the season during which data were acquired. This is consistent with the results of the data re-analysis presented here. They suggest that the sensitivity to high melanopic compared to low melanopic light is reduced in summer compared to winter, as indicated by the reduced impact of melanopsin activation on sleep latency and melatonin suppression during the summer months. Further support for enhanced melatonin suppression in winter comes from Owen et al. and Higuchi et al. [17,91]. The study by Higuchi et al. [91] showed a greater melatonin suppression 2 h after the start of light exposure (1000 lx, 4200 K) in winter (66.6 ± 18.4%) than in summer (37.2 ± 33.2%). The ambient light dose participants received from rising until going to bed in summer was approximately twice as high in summer as in winter. Nathan et al. [92] could, however, not find seasonal differences in melatonin suppression when participants were exposed to light (200 lx) for one hour. Münch et al. [7] compared melanopsin-mediated light responses in summer and winter and found a seasonal variation in the Post-Illumination Pupil Response (PIPR) in pseudophakes indicating seasonal variations. The PIPR is formed by intrinsically photosensitive retinal ganglion cells (ipRGCs) and is, therefore, a proxy for melanopsin system sensitivity [93]. However, they found no differences in melatonin suppression after 30 min of light exposure at 400 lx. The authors concluded that their light exposure may have been too short and that the age of their participants (mean: 67 years) might have influenced melatonin suppression [94]. In contrast, in our protocol, young participants (19–35 years, mean: 25 years) were exposed for a longer time (3.5 h) but to relatively low light levels (<90 lx). Our re-analysis is, to the best of our knowledge, the first to suggest that specifically melanopsin-mediated melatonin suppression may be more pronounced in winter than in summer.

### 3.3. Seasonal Variation of Light-Induced Changes in Melatonin Circadian Phase

In addition to the more acute effects of prior light history on sensitivity to evening melatonin suppression, it may also have affected the circadian phase. It is well established that 24 h light exposure patterns determine human circadian entrainment, with morning light advancing and evening light delaying human circadian melatonin rhythms [95]. Several studies have reported a phase delay in winter compared to summer in circadian melatonin rhythms measured either in saliva, plasma or urine in different locations such as Australia, Siberia and Central Europe [96,97,98,99]. However, because our participants’ visits took place within two weeks at their habitual bedtimes, such effects would have been masked. Moreover, our phase estimate was not based on a 24 h assessment of the circadian profile of melatonin, but only on melatonin onset, which has been masked by the acute effect of light.

However, the aim here was to describe whether the effect of melanopic high versus melanopic low light on the phase of the melatonin rise varies seasonally rather than to describe fundamental seasonal differences in the timing of the evening melatonin rise. We found that in both summer and winter, the onset of melatonin relative to bedtime was significantly delayed in the high melanopic compared to the low melanopic condition.

### 3.4. Seasonal Variation in Sleep Architecture and Its Modification by Light

While there have been studies of the seasonal effect on subjective and actigraphy-assessed sleep duration and timing, with longer sleep duration and earlier bedtimes in winter than in summer [100,101], there is limited research on seasonal changes in sleep architecture as assessed by PSG. Altogether, these studies suggest that the received dose of daylight may well affect sleep architecture. More specifically, in a laboratory study, a shorter photoperiod (10 h vs. 16 h) was shown to result in longer sleep times [102]. This is consistent with Seidler et al. [6] who found longer total sleep time during winter compared to summer using PSG. Furthermore, there is evidence for seasonal effects on REM sleep [6,103,104], but the direction of these effects is controversial. REM sleep was found to be shorter during autumn than in spring [6,104] and during winter than during summer [104], whereas Kohsaka et al. [103] found more REM sleep (about 30 min) in winter compared to spring. As REM sleep is under strong circadian control [105,106,107], simultaneous assessment of the circadian melatonin phase and PSG is recommended for studies investigating seasonal effects of REM sleep. Kohsaka et al. also found that sleep timing was 1–1.5 h later in winter than in summer, while Seidler et al. [6] did not report a change in sleep timing. Neither study reported changes in sleep latency across seasons. Importantly, neither of these studies investigated the overall effect of evening light exposure on sleep, but they do suggest that the amount of light during the day may have influenced sleep architecture. This is the first evidence that the light sensitivity of the human melanopsin system and its effect on sleep latency depends on the season and possibly on the individual’s previous light history. Our results suggest that in summer, the effects of high melanopic light compared to low melanopic light are not as pronounced as in winter. Therefore, collecting data only in winter, as many studies have done, may have led to an overestimation of the effects on sleep latency and, thus, reduced replicability when the timing of data collection is unknown.

### 3.5. Suggestions for Improving the Reproducibility of Studies Investigating Non-Visual Effects of Light in Humans

**Figure 4:**
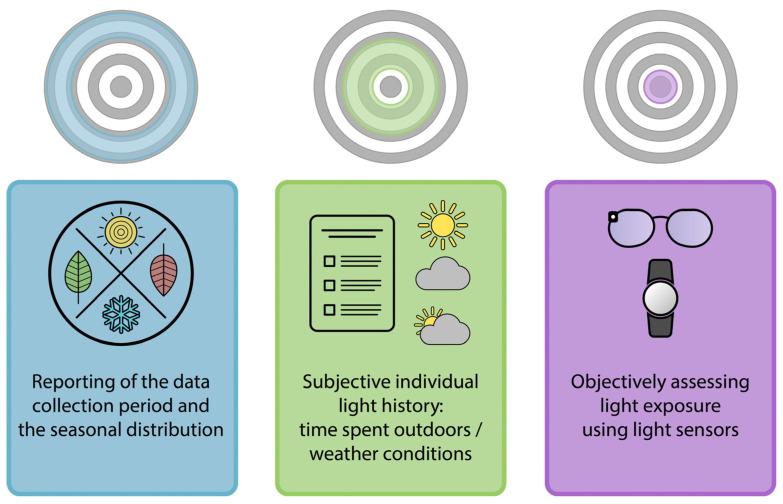
Schematic illustration of suggestions for reporting season and light history and their accuracy in predicting individual light history.

Here, we would like to add some suggestions to the existing guidelines for reporting light exposure in human chronobiology and sleep research experiments [108]:

1. Studies investigating not only the non-visual effects of light in humans but also any endpoint under potential seasonal influence should report the season, seasonal distribution, and time of day of the respective measurement.
2. The assessment and reporting of subjective individual light history (i.e., time spent outdoors) is a relatively simple method, together with weather conditions. The assessment should also include the time of day when outdoor activities took place. In our study, for example, subjectively reported time spent outdoors showed relatively small differences in summer and winter. This is not unlikely, given that most of our participants were students and the study was conducted during the COVID-19 pandemic.
3. Taking into account the solar irradiance from local weather stations and combining this with individual time and duration spent outdoors improves the prediction of individual light history, and, for example, our study showed significant differences between summer and winter months. The most accurate is the objective assessment of individual light history by light sensors, which can be worn on different parts of the body (e.g., wrist-worn, on eye-level attached to spectacle frames or worn at the chest), the more advanced of which also provide spectral characterization of light (for an overview, see [109]) (Figure 4).
4. One strategy for eliminating the effects of prior light history, if desired, is to keep participants in the laboratory on study days and control the light situation, thus reducing variance by eliminating the bias caused by variations in prior light exposure. In most constant routine studies of the effects of evening light on melatonin (e.g., [38]), illuminance was drastically reduced compared to daylight (<100 lx vs. 1000 lx (overcast day)—100,000 lx (direct sunlight)) when participants remained in the laboratory throughout the experimental day. It should be noted, however, that the ecological validity of these strictly light-controlled studies is compromised by the fact that reducing light exposure during the day increases sensitivity to evening light [15,54]. This should be kept in mind when comparing the results of different study designs.

## 4. Conclusions and Summary

Depending on latitude, the changes in photoperiod are immense and may have major effects on human physiology and behavior. However, despite the use of artificial light in modern lifestyles to override seasonal effects in photoperiods, it is not clear how sensitive we still are to photoperiodic changes at a given latitude. For this reason, studies of the non-visual effects of light on circadian melatonin and sleep in humans need to include data collection and recording dates.

Unfortunately, sleep and circadian studies often involve small numbers of people being studied. As a result, seasonality is often excluded from data analyses or only collected in winter. Here, we report that sensitivity to melanopic irradiance would have been more pronounced if we had conducted our study only in winter months when the photoperiod is shorter. Therefore, the results of studies that only collect data in one season cannot be extrapolated to the whole year, as this may lead to an overestimation of study results in winter and vice versa in summer.

In summary, a relatively simple way to improve the reproducibility of research results is to provide information on the period of data collection and the seasonal distribution (e.g., the number of observations per month). Secondly, any information on subjective light behavior (i.e., time spent outdoors) and light changes recorded by a light logger to quantify a person’s light history would greatly improve the ability to disentangle the effects of photic memory on human circadian physiology and sleep and improve reproducibility among studies.

## 5. Materials and Methods

### 5.1. Review

Criteria for eligibility were that the studies included human participants, information on the spectral power distribution or illuminance levels and specified the duration of light exposure. Moreover, the timing of light exposure had to have occurred in the evening within the time interval from 5:30 p.m. to 2 a.m. prior to nighttime sleep and lasted for at least 30 min. We only included original research in the English language and laboratory studies. There were no restrictions regarding the study design or prior light history, sex or age of the participants. Letters to the editor, conference abstracts and literature reviews were excluded (according to [110]).

#### 5.1.1. Search Melatonin Studies

The search was performed on 9 June 2023, in PubMed and Web of Science. The search string included the items (Light [All Fields] AND Melatonin [All Fields]) AND Evening [All Fields]). The search on PubMed yielded 448 articles, and the search on Web of Science yielded 721 articles. After removing duplicates, the articles were screened by IS, OS and CB for exclusion from studies.

#### 5.1.2. Search Polysomnographically Assessed Sleep

We only included studies that assessed sleep by polysomnography in a laboratory setting. Studies observing the evening light-dependent effects on polysomnographically assessed night-time sleep are limited. We used 18 studies selected in our meta-analysis (Cajochen, 2022) and added the literature published between 22 January 2021, and 28 May 2023. Therefore, the PubMed and Web of Science search included the following items according to [110]: ((Light [title] OR Lighting [title]) AND Sleep [title]) AND humans [mesh]). We excluded three studies used by Cajochen et al. [110] because two studies observed light exposure during the night [111,112] and one study showed light exposure throughout the day [113]. The search on PubMed yielded 104 articles, and the search on Web of Science yielded 99 articles. After removing duplicates, 105 articles were screened. Only three additional papers could be included. The most common reasons for exclusion were that there was no polysomnography, no laboratory study, the light exposure was not in the evening, or there was no original data.

#### 5.1.3. Collected Data

We collected information on the light timing, duration, the period of data collection, season and any information about seasonal distributions. The light history was classified as controlled when the participant spent the whole day in the laboratory since already morning light can have phase-advancing effects [114] and is otherwise considered uncontrolled.

### 5.2. Seasonal Dependent Data

We used the melatonin and sleep latency data published by Schöllhorn et al. [12]. Seventy-two young (18–35 years; Intensity 1: 24.5 ± 3.8 years; Intensity 2: 25.4 ± 5.5 years; Intensity 3: 24.3 ± 4.3 years; Intensity 4: 24.7 ± 3.5 years) healthy male participants have been included in the study. The study investigated the influence of melanopic irradiance on melatonin and polysomnographically recorded sleep latency within four light-intensity groups (*n* = 18 participants per group). Within each light intensity group (27–284 cd/m^2^), there was a high melanopic and a low melanopic light condition (within comparison), which showed an approximately 3-fold difference in melanopic irradiance (for details, see [12] or Supplementary Tables S3 and S4 with light characteristics). After entering the laboratory 7 h before habitual bedtime, there were two periods of dark adaptation and one dim light period (~2 h in total) before participants were exposed to the different light conditions. Four hours before habitual bedtime, participants were exposed to the different light conditions for about 3.5 h.

The study was carried out between December 2019 and July 2021. To assess whether there are differences in evening sensitivity to high melanopic light between seasons, data from all four light intensity groups were combined. As many previous studies were conducted only during the winter months, light sensitivity in summer (April to September) and winter (October to March) were analyzed separately using Linear Mixed Models. Approximately the same number of appointments were conducted in summer (*n* = 73) and winter (*n* = 71), and the number of appointments in summer and winter at the two lowest (winter: *n* = 35, summer: *n* = 37) and two highest luminance levels (winter: *n* = 36, summer: *n* = 36) was similar. All statistical analyses were conducted in R (Version 4.1.1, R Core Team, 2021). For all variables, Light Condition (HM and LM) was included as a fixed effect and repeated measures per participant were modeled as a random intercept. LMMs were followed by an ANOVA (Type III) function. We used log-transformed sleep latency values for our analyses. As an effect size measure, omega squared (ω^2^) was calculated using the effect size package. It can be interpreted as follows: small effect: ω^2^ ≥ 0.01, medium effect: ω^2^ ≥ 0.06, large effect: ω^2^ ≥ 0.14. A *p*-value < 0.05 was considered to indicate statistical significance.

## Data Availability

Data on Melatonin and Sleep Latency can be found at Schöllhorn et al. [12]. The datasets are available from the corresponding author on reasonable request.

## Supplementary Materials

The following supporting information can be downloaded at: https://www.mdpi.com/article/10.3390/clockssleep5040044/s1, Table S1: Description of the included studies on evening light exposure on melatonin concentration; Table S2: Description of the included studies on the effect of evening light exposure on sleep as assessed by polysomnography; Table S3: Overview of luminances, illuminances, chromaticity coordinates and irradiance-derived α-opic responses; Table S4: α-opic equivalent daylight (D65) illuminances, mEDI ratios and contrasts. Figure S1: Time spent outdoors prior to the arrival at the laboratory on experimental days in summer and winter. Figure S2: Boxplots of light effects depending on the season for the four light intensity groups. Reference [115] is cited in the supplementary materials.
