# Supplementary material for: Seasonal Variation in the Responsiveness of the Melanopsin System to Evening Light: Why We Should Report Season When Collecting Data in Human Sleep and Circadian Studies
Source: Clocks Sleep. 2023 Nov 1;5(4):651–66. doi: 10.3390/clockssleep5040044 (PMC10660855; doi:10.3390/clockssleep5040044)
Supplement: Supplementary file 1 [file clockssleep-05-00044-s001.zip › clockssleep-2516134-supplementary.pdf]

Supplementary Information

Seasonal variation in the responsiveness of the melanopsin system to evening light: why we should report season when collecting data in human sleep and circadian studies

Isabel Schöllhorn[1,2], Oliver Stefani[1,2,3], Christine Blume[1,2], Christian Cajochen[1,2]

1 Centre for Chronobiology, Psychiatric Hospital of the University of Basel, Basel, Switzerland

2 Research Platform Molecular and Cognitive Neurosciences (MCN), University of Basel, Basel, Switzerland

3 Lucerne University of Applied Sciences and Arts, Technikumstrasse 21, 6048 Horw, Switzerland

Name	Year	Light Timing	Duration	Period data collection	Information about season	Season	In the lab during experimental days	Light History assessed/in lab during experimental day
**Chinoy et al. [1]**	2018	18:00-21:00	3 h	/	0	NA	1	~89 lux-150 lx
**Rangtell et al. [2]**	2016	21:00-23:00	2 h	/	0	NA	0	/
**Santhi et al. [3]**	2012	4.25 HPB	4 h	Oct.-Feb.	1	Winter	0	Actigraphy
**Figueiro et al. [4]**	2014	23:00-00:30	1.5 h	/	0	NA	0	/
**Kräuchi et al. [5]**	1997	21:00–24:00	3 h	Oct.-May	1	mostly Winter	0	/
**Hartmann et al. [6]**	2019	17:45-23:37		Sept.- Feb.	1	mostly Winter	0	/
**Thompson et al. [7]**	2015	21:45-22:15	0.5 h	Nov.-Apr.	1	mostly Winter	0	/
**Lack et al. [8]**	1993	20:00-24:00	4 h	/	1	Winter	0	100 lux and uncontrolled bright light
**Souman et al. [9]**	2018	2-1 h HBT	1 h	Sept.-Oct.	1	Summer and Winter	0	Actigraphy
**Knaier et al. [10]**	2017	median 21:17 PM;17 h after the individual MSFsc	1 h	Apr-2014 – Apr. 2015	1	Summer and Winter	0	Not reported, but Actigraphy
**Höhn et al. [11]**	2021	~2.5 HPB	3x25min	Oct. – Dec.	1	Winter	0	/
**Kennaway et al [12]**	1987	19:00-02:00	7 h	Mar.	1	Winter	0	/
**Wahnschaffe et al. [13]**	2013	1 HPB	1 h	Feb.	1	Winter	0	/
**Chellappa et al. [14]**	2011	21:30-23:00	2 h	Jan. – Mar.	1	Winter	0	/
**Spitschan et al. [15]**	2019	2.5 - 0.5 HPB	2 h	Nov. – June	1	Summer and Winter	0	/
**Ritter et al. [16]**	2020	23:00-23.30	0.5 h	Nov. –Mar.	1	Winter	0	/
**Lasko et al. [17]**	1999	00:00-02:00	2 h	/	0	NA	0	/
**Schöllhorn et al. [18]**	2023	4 HBT	3.5 h	Dec. 2019 – July 2021	1	Summer and Winter	0	Subjective
**Schmidt et al. [19]**	2018	2 h prior sleep time	2 h	/	0	NA	0	/

**Weng et al. [20]**	2022	20:00-23:00 or 20:45-23-45	3 h	/	0	NA	0	/
**Chang et al. [21]**	2012	0.5-4.5 h after melatonin onset	0.2, 1, 2.5, 4	/	0	NA	1	below 3 lx
**Schmidt et al. [22]**	2021	20:45-22:15	1.5 h	Oct. 2019 – Dec. 2020	1	Summer and Winter	0	/
**Cajochen et al. [23]**	2005	13.5 h after wake up	2 h	/		NA	0	/
**Phillips et al. [24]**	2019	4 HPB	5 h	/	0	NA	0	Actigraphy
**Allen et al. [25]**	2018	18:00-23:00	5 h	Apr. – July	1	Summer	0	/
**Chang et al. [26]**	2015	18:00-22:00	4 h	/	0	NA	1	90 lx
**Wirz-Justice et al. [27]**	2004	21:00–24:00	3 h	/	0	NA	1	room light, after 2pm <10lx
**Green et al. [28]**	2017	21:00-23:00	2 h	/	0	NA	0	/
**Cajochen et al. [29]**	2006	22:00-24:00	2 h	/	0	NA	0	/
**Cajochen et al. [30]**	2011	4 HPB	5 h	End of Sep. – Dec.	1	mostly Winter	0	/
**Jones et al. [31]**	2018	20:00-22:00	2 h	/	0	NA	0	/
**Jo et al. [32]**	2018	17:30-24:00	6.5 h	/	0	NA	0	/
**Harada et al. [33]**	2004	19:25-22:30 afterwards 250 lx until 23:40	3 h	5th Jan.; Winter	1	Winter	0	350-500 lx driving in the car before the experiment, 6000–7500 lux at the eye level during 12:30–13:30 and 14:00–14:50, 250 lx till 16:30,
**Bunnel et al. [34]**	1992	2 HPB	2 h	/	0	NA	0	/
**Knaier et al. [35]**	2017	17 h after the individual MSFsc	1 h	Apr. 2014 – Apr. 2015	1	Summer and Winter	0	/
**Wright et al. [36]**	2001	24:00-02:00	2 h	Autumn and Winter	1	Mostly Winter	0	/
**Te Kulve et al. [37]**	2019	18:30 -21:00; 22:30-23:30	2.5 h or 3.5 h	/	0	NA	0	/
**Nagare et al. [38]**	2019	23:00-01:00	2 h	June	1	Summer	0	/
**Muench et al. [39]**	2015	17 h after habitual wake-time	2 h	/	0	NA	0	/

**Green et al. [40]**	2018	21:00-23:00		/	0	NA	0	/
**Moderie et al. [41]**	2017	1 hour after bedtime	1.5 h	/	0	NA		/
**Lovato et al. [42]**	2016	00:00-01:00 and 01:00-02:00 h	1 h	Aug. 2013–Jan. 2014	1	Summer and Winter		/
**van der Lely et al. [43]**	2015	3 HPB	3 h	/	0	NA	0	Light logger: Luxblick
**Chellappa et al. [44]**	2019	13.5 h after wake up	2 h	Feb. 2012–Apr. 2014	1	Summer and Winter	0	/
**Nowozin et al [45]**	2017	1 HPB	0.5 h	fall and winter, Feb. –Apr., Oct. –Feb.	1	mostly Winter	0	/

Table S1 Description of the included studies on evening light exposure on melatonin concentration. Abbreviations: January (Jan.), February (Feb.), March (Mar.), April (Apr.), May (May), June (Jun.), July (Jul.), August (Aug.), September (Sep.), October (Oct.), November (Nov.), December (Dec.), Hours Prior Bedtime (HPB).

Name	Year	Light Timing	Duration [min]	Period data collection	Information about season	Season	In the lab during experimental days	Light History assessed/in lab during experimental day
**Saletu [46]**	1986	17:00-21:00 and 06:00-09:00	7 h	/	0	/	1	/
**Drennan [47]**	1989	18:00-21:00 3 times	3 h	Sep. – Mar	1	mostly Winter	0	/
**Dawson [48]**	1991	24:00-04:00 3 times	4 h	/	0	/	0	/
**Cajochen [49]**	1992	21:00-24:00	3 h	Feb. – Mar	1	Winter	0	/
**Dumont [50]**	1997	18:30-23:30 3 times	5 h	May – Sep.	1	Summer	1	/
**Cajochen [51]**	1998	21:00-24:00	3 h	/	0	/	0	Sunglasses before lab, controlled after 10 a.m.
**Gordijn [52]**	1999	18:00-21:00 24 hour prior	3 h 24 hours prior	Throughout the year	1	Summer and Winter	0	/
**Komada [53]**	2000	just prior sleep	40 min	/	0	/	0	/
**Burgess [54]**	2001	ca. 2 h prior sleep	4.5 h	/	0	/	0	/
**Kozaki [55]**	2005	19:30-02:00	6.5 h	Jul. – Nov.	1	Summer and Winter	0	/
**Münch [56]**	2006	21:30-23:30	2 h	/	0	/	0	/
**Cajochen [57]**	2008	22:00-24:00	2 h	/	0	/	/	Avoid sunlight, Dim light (2-8 PM)

**Münch [58]**	2011	21:00-23:00	2 h	/	0	/	1	dim (~1 μW/ cm²)
**Santhi [3]**	2012	4.25 HPB	4 h	Oct. – Feb.	1	Winter	0	Actigraphy
**Chellappa [59]**	2013	Until 45 min prior sleep	2 h	Jan.–Mar.	1	Winter	0	/
**Chang [26]**	2015	18:00-22:00	4 h	/	0	/	1	90 lx
**Rangtell [2]**	2016	21:00-23:00	2 h	/	0	/	0	/
**Green [28]**	2017	21:00-23:00	2 h	/	0	/	0	/
**Hilditch [60]**	2022	After 10 consecu-tive 30-s epochs SWS	1 h	/	0	/	0	/
**Vethe [61]**	2022	18:00-23:00	5 h	Sep. – Oct.	1	Summer and Winter	0	/
**Schöllhorn et al. [18]**	2023	4 HPB	3.5 h	Dec. 2019-July 2021	1	Summer and Winter	0	Subjective

Table S2 Description of the included studies on the effect of evening light exposure on sleep as assessed by polysomnography. Abbreviations: January (Jan.), February (Feb.), March (Mar.), April (Apr.), May (May), June (Jun.), July (Jul.), August (Aug.), September (Sep.), October (Oct.), November (Nov.), December (Dec.), Hours Prior Bedtime (HPB).

**Condition**	**LM 1**	**HM 1**	**LM 2**	**HM 2**	**LM 3**	**HM 3**	**LM 4**	**HM 4**
**Luminance [cd/m²]**	27.43	27.41	62.93	61.39	135.03	133	283.74	284.5
**Illuminance [lx]**	8.61	7.18	19.45	15.94	41.96	34.36	88.63	73.66
**CIE 1964 $x_{10}y_{10}$ chromaticity (x_{10})**	0.34	0.33	0.33	0.34	0.34	0.34	0.34	0.34
**CIE 1964 $x_{10}y_{10}$ chromaticity (y_{10})**	0.33	0.33	0.34	0.33	0.34	0.33	0.34	0.33
**S-cone-opic irradiance (mW · m^{-2})**	6.40	6.22	13.87	13.72	29.58	29.71	60.21	62.00
**M-cone-opic irradiance (mW · m^{-2})**	11.19	11.74	25.52	25.43	55.08	54.79	116.58	115.12
**L-cone-opic irradiance (mW · m^{-2})**	13.32	13.50	29.95	30.09	64.90	64.78	138.10	138.86
**Rhodopic irradiance (mW · m^{-2})**	6.87	18.70	16.47	40.39	38.09	86.81	87.00	180.89

**Melanopic irradiance (mW · m⁻²)**	4.91	20.11	11.70	43.51	27.46	93.27	64.17	193.62

Table S3: Overview of luminances, illuminances, chromaticity coordinates and irradiance-derived α-opic responses. The values have been calculated using the luox app.[62]

Condition	LM 1	HM 1	LM 2	HM 2	LM 3	HM 3	LM 4	HM 4
**S-cone-opic EDI [lx]**	7.83	7.61	16.97	16.79	36.19	36.36	73.67	75.86
**M-cone-opic EDI [lx]**	7.69	8.07	17.53	17.47	37.83	37.63	80.08	79.08
**L-cone-opic EDI [lx]**	8.18	8.29	18.39	18.47	39.84	39.77	84.78	85.25
**Rhodopic EDI [lx]**	4.74	12.90	11.36	27.86	26.28	59.88	60.01	124.78
**mEDI [lx]**	3.70	15.17	8.82	32.81	20.70	70.33	48.39	146.00
**Ratio** (HM/LM)	4.1		3.72		3.40		3.02	
**Contrast** (HM-LM)/LM [%]	310		272		240		202	

Table S4: α-opic equivalent daylight (D65) illuminances, mEDI ratios and contrasts. α-opic equivalent daylight (D65) illuminances have been calculated using the luox app.[62]

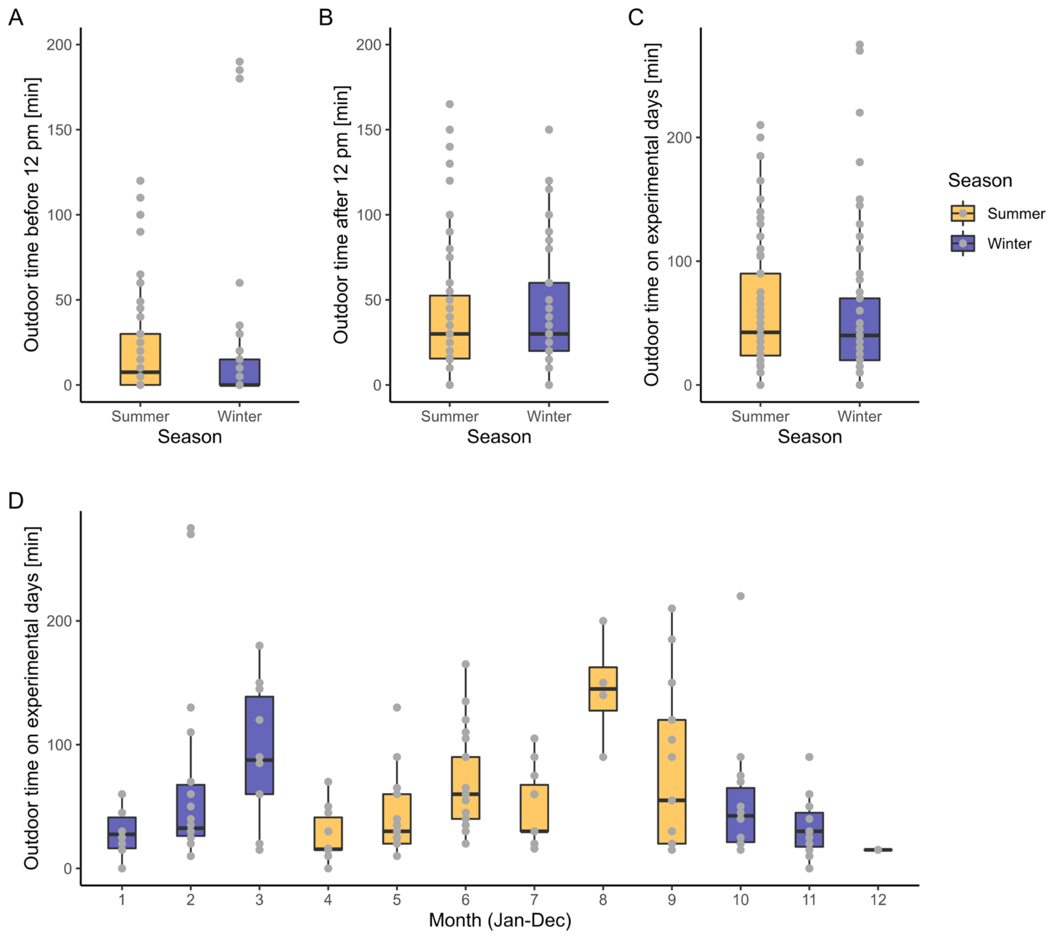

Figure S1. Time spent outdoors prior to the arrival at the laboratory on experimental days in summer and winter: A Before 12 am; B After 12 am; C On experimental days; D On experimental days for each season. The coloured boxes indicate whether the data was collected in summer (yellow) or winter (purple). The lower and upper hinges of each box correspond to the first and third quartiles (the 25th and 75th percentiles). The black horizontal bar within each box refers to the median. The upper (lower) whisker extends from the hinge to the largest (lowest) value no further than 1.5 * the inter-quartile range (IQR). The individual values are highlighted in grey.

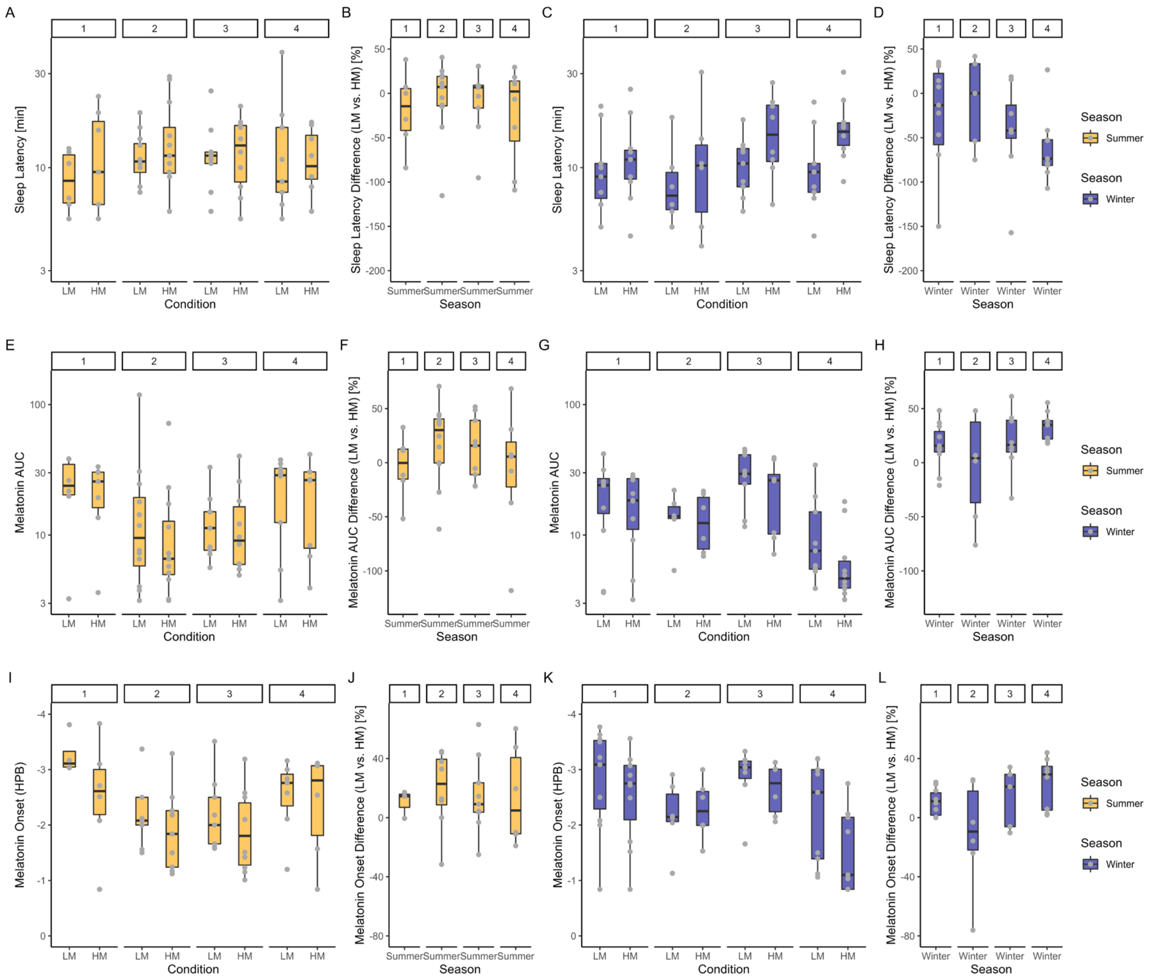

Figure S2. Boxplots of light effects depending on the season for the four light intensity groups: A-D Sleep Latency in minutes on a log scale; E-H Melatonin Area Under the Curve (AUC); I-L Melatonin Onset in hours prior to bedtime (HPB). The difference [%] corresponds to (LM-HM)/LM. The coloured boxes indicate whether the data was collected in summer (yellow) or winter (purple). The lower and upper hinges of each box correspond to the first and third quartiles (the 25th and 75th percentiles). The black horizontal bar within each box refers to the median. The upper (lower) whisker extends from the hinge to the largest (lowest) value no further than 1.5 * the inter-quartile range (IQR). The individual values are highlighted in grey. Abbreviations: HPB: Hours prior bedtime, LM: Low Melanopic Condition; HM High Melanopic Condition.

References

1. Chinoy ED, Duffy JF, Czeisler CA. Unrestricted evening use of light-emitting tablet computers delays self-selected bedtime and disrupts circadian timing and alertness. Physiol Rep. 2018;6: e13692. doi:10.14814/phy2.13692

2. Rångtell FH, Ekstrand E, Rapp L, Lagermalm A, Liethof L, Búcaro MO, et al. Two hours of evening reading on a self-luminous tablet vs. reading a physical book does not alter sleep after daytime bright light exposure. Sleep Medicine. 2016;23: 111–118. doi:10.1016/j.sleep.2016.06.016

3. Santhi N, Thorne HC, van der Veen DR, Johnsen S, Mills SL, Hommes V, et al. The spectral composition of evening light and individual differences in the suppression of melatonin and delay of sleep in humans: Artificial evening light suppresses melatonin and delays sleep. Journal of Pineal Research. 2012;53: 47–59. doi:10.1111/j.1600-079X.2011.00970.x

4. Figueiro MG, Wood B, Plitnick B, Rea MS. The impact of watching television on evening melatonin levels: Impact of watching television on evening melatonin. Jnl Soc Info Display. 2013;21: 417–421. doi:10.1002/jsid.200

5. Kräuchi K, Cajochen C, Danilenko KV, Wirz-Justice A. The hypothermic effect of late evening melatonin does not block the phase delay induced by concurrent bright light in human subjects. Neuroscience Letters. 1997;232: 57–61. doi:10.1016/S0304-3940(97)00553-3

6. Hartmann M, Pelzl MA, Kann PH, Koehler U, Betz M, Hildebrandt O, et al. The effects of prolonged single night session of videogaming on sleep and declarative memory. PLoS One. 2019;14: e0224893. doi:10.1371/journal.pone.0224893

7. Thompson A, Jones H, Marqueze E, Gregson W, Atkinson G. The Effects of Evening Bright Light Exposure on Subsequent Morning Exercise Performance. Int J Sports Med. 2014;36: 101–106. doi:10.1055/s-0034-1389970

8. Lack L, Wright H. The effect of evening bright light in delaying the circadian rhythms and lengthening the sleep of early morning awakening insomniacs. Sleep. 1993;16: 436–443. doi:10.1093/sleep/16.5.436

9. Souman JL, Borra T, de Goijer I, Schlangen LJM, Vlaskamp BNS, Lucassen MP. Spectral Tuning of White Light Allows for Strong Reduction in Melatonin Suppression without Changing Illumination Level or Color Temperature. J Biol Rhythms. 2018;33: 420–431. doi:10.1177/0748730418784041

10. Knaier R, Schäfer J, Rossmeissl A, Klenk C, Hanssen H, Höchsmann C, et al. Prime Time Light Exposures Do Not Seem to Improve Maximal Physical Performance in Male Elite Athletes, but Enhance End-Spurt Performance. Front Physiol. 2017;8: 264. doi:10.3389/fphys.2017.00264

11. Höhn C, Schmid SR, Plamberger CP, Bothe K, Angerer M, Gruber G, et al. Preliminary Results: The Impact of Smartphone Use and Short-Wavelength Light during the Evening on Circadian Rhythm, Sleep and Alertness. Clocks & Sleep. 2021;3: 66–86. doi:10.3390/clockssleep3010005

12. Kennaway DJ, Earl CR, Shaw PF, Royles P, Carbone F, Webb H. Phase Delay of the Rhythm of 6-Sulphatoxy Melatonin Excretion by Artificial Light. J Pineal Res. 1987;4: 315–320. doi:10.1111/j.1600-079X.1987.tb00869.x

13. Wahnschaffe A, Haedel S, Rodenbeck A, Stoll C, Rudolph H, Kozakov R, et al. Out of the Lab and into the Bathroom: Evening Short-Term Exposure to Conventional Light Suppresses Melatonin and Increases Alertness Perception. IJMS. 2013;14: 2573–2589. doi:10.3390/ijms14022573

14. Chellappa SL, Steiner R, Blattner P, Oelhafen P, Götz T, Cajochen C. Non-Visual Effects of Light on Melatonin, Alertness and Cognitive Performance: Can Blue-Enriched Light Keep Us Alert? Herzog MH, editor. PLoS ONE. 2011;6: e16429. doi:10.1371/journal.pone.0016429

15. Spitschan M, Lazar R, Yetik E, Cajochen C. No evidence for an S cone contribution to acute neuroendocrine and alerting responses to light. Curr Biol. 2019;29: R1297–R1298. doi:10.1016/j.cub.2019.11.031

16. Ritter P, Wieland F, Skene DJ, Pfennig A, Weiss M, Bauer M, et al. Melatonin suppression by melanopsin-weighted light in patients with bipolar I disorder compared to healthy controls. jpn. 2020;45: 79–87. doi:10.1503/jpn.190005

17. Lasko TA, Kripke DF, Elliot JA. Melatonin Suppression by Illumination of Upper and Lower Visual Fields. J Biol Rhythms. 1999;14: 122–125. doi:10.1177/074873099129000506

18. Schöllhorn I, Stefani O, Lucas RJ, Spitschan M, Slawik HC, Cajochen C. Melanopic irradiance defines the impact of evening display light on sleep latency, melatonin and alertness. Commun Biol. 2023;6: 228. doi:10.1038/s42003-023-04598-4

19. Schmidt C, Xhrouet M, Hamacher M, Delloye E, LeGoff C, Cavalier E, et al. Light exposure via a head-mounted device suppresses melatonin and improves vigilant attention without affecting cortisol and comfort: Head-mounted light, melatonin, vigilance, & comfort. Psych J. 2018;7: 163–175. doi:10.1002/pchj.215

20. Weng M, Schöllhorn I, Kazhura M, Cardini BB, Stefani O. Impact of Evening Light Exposures with Different Solid Angles on Circadian Melatonin Rhythms, Alertness, and Visual Comfort in an Automotive Setting. Clocks & Sleep. 2022;4: 607–622. doi:10.3390/clockssleep4040047

21. Chang A-M, Santhi N, St Hilaire M, Gronfier C, Bradstreet DS, Duffy JF, et al. Human responses to bright light of different durations: Light DRC in humans. The Journal of Physiology. 2012;590: 3103–3112. doi:10.1113/jphysiol.2011.226555

22. Schmid SR, Höhn C, Bothe K, Plamberger CP, Angerer M, Pletzer B, et al. How Smart Is It to Go to Bed with the Phone? The Impact of Short-Wavelength Light and Affective States on Sleep and Circadian Rhythms. Clocks & Sleep. 2021;3: 558–580. doi:10.3390/clockssleep3040040

23. Cajochen C, Münch M, Kobialka S, Kräuchi K, Steiner R, Oelhafen P, et al. High Sensitivity of Human Melatonin, Alertness, Thermoregulation, and Heart Rate to Short Wavelength Light. The Journal of Clinical Endocrinology & Metabolism. 2005;90: 1311–1316. doi:10.1210/jc.2004-0957

24. Phillips AJK, Vidafar P, Burns AC, McGlashan EM, Anderson C, Rajaratnam SMW, et al. High sensitivity and interindividual variability in the response of the human circadian system to evening light. Proc Natl Acad Sci USA. 2019;116: 12019–12024. doi:10.1073/pnas.1901824116

25. Allen AE, Hazelhoff EM, Martial FP, Cajochen C, Lucas RJ. Exploiting metamerism to regulate the impact of a visual display on alertness and melatonin suppression independent of visual appearance. Sleep. 2018;41. doi:10.1093/sleep/zsy100

26. Chang A-M, Aeschbach D, Duffy JF, Czeisler CA. Evening use of light-emitting eReaders negatively affects sleep, circadian timing, and next-morning alertness. Proc Natl Acad Sci USA. 2015;112: 1232–1237. doi:10.1073/pnas.1418490112

27. Wirz-Justice A, Krauchi K, Cajochen C, Danilenko KV, Renz C, Weber JM. Evening melatonin and bright light administration induce additive phase shifts in dim light melatonin onset. J Pineal Res. 2004;36: 192–194. doi:10.1111/j.1600-079X.2004.00117.x

28. Green A, Cohen-Zion M, Haim A, Dagan Y. Evening light exposure to computer screens disrupts human sleep, biological rhythms, and attention abilities. Chronobiology International. 2017;34: 855–865. doi:10.1080/07420528.2017.1324878

29. Cajochen C, Jud C, Münch M, Kobialka S, Wirz-Justice A, Albrecht U. Evening exposure to blue light stimulates the expression of the clock gene *PER2* in humans. European Journal of Neuroscience. 2006;23: 1082–1086. doi:10.1111/j.1460-9568.2006.04613.x

30. Cajochen C, Frey S, Anders D, Späti J, Bues M, Pross A, et al. Evening exposure to a light-emitting diodes (LED)-backlit computer screen affects circadian physiology and cognitive performance. Journal of Applied Physiology. 2011;110: 1432–1438. doi:10.1152/japplphysiol.00165.2011

31. Jones MJ, Peeling P, Dawson B, Halson S, Miller J, Dunican I, et al. Evening electronic device use: The effects on alertness, sleep and next-day physical performance in athletes. Journal of Sports Sciences. 2018;36: 162–170. doi:10.1080/02640414.2017.1287936

32. Jo H, Park HR, Choi SJ, Lee S-Y, Kim SJ, Joo EY. Effects of Organic Light-Emitting Diodes on Circadian Rhythm and Sleep. Psychiatry Investig. 2021;18: 471–477. doi:10.30773/pi.2020.0348

33. Harada T. Effects of evening light conditions on salivary melatonin of Japanese junior high school students. J Circadian Rhythms. 2005;2: 4. doi:10.1186/1740-3391-2-4

34. Bunnell DE, Treiber SP, Phillips NH, Berger RJ. Effects of evening bright light exposure on melatonin, body temperature and sleep. Journal of Sleep Research. 1992;1: 17–23. doi:10.1111/j.1365-2869.1992.tb00003.x

35. Knaier R, Schäfer J, Rossmeissl A, Klenk C, Hanssen H, Höchsmann C, et al. Effects of bright and blue light on acoustic reaction time and maximum handgrip strength in male athletes: a randomized controlled trial. Eur J Appl Physiol. 2017;117: 1689–1696. doi:10.1007/s00421-017-3659-0

36. Wright HR, Lack LC. EFFECT OF LIGHT WAVELENGTH ON SUPPRESSION AND PHASE DELAY OF THE MELATONIN RHYTHM. Chronobiology International. 2001;18: 801–808. doi:10.1081/CBI-100107515

37. Te Kulve M, Schlangen LJM, Van Marken Lichtenbelt WD. Early evening light mitigates sleep compromising physiological and alerting responses to subsequent late evening light. Sci Rep. 2019;9: 16064. doi:10.1038/s41598-019-52352-w

38. Nagare R, Plitnick B, Figueiro M. Does the iPad Night Shift mode reduce melatonin suppression? Lighting Research & Technology. 2019;51: 373–383. doi:10.1177/1477153517748189

39. Münch M, Léon L, Collomb S, Kawasaki A. Comparison of acute non-visual bright light responses in patients with optic nerve disease, glaucoma and healthy controls. Sci Rep. 2015;5: 15185. doi:10.1038/srep15185

40. Green A, Cohen-Zion M, Haim A, Dagan Y. Comparing the response to acute and chronic exposure to short wavelength lighting emitted from computer screens. Chronobiology International. 2018;35: 90–100. doi:10.1080/07420528.2017.1387555

41. Moderie C, Van Der Maren S, Dumont M. Circadian phase, dynamics of subjective sleepiness and sensitivity to blue light in young adults complaining of a delayed sleep schedule. Sleep Medicine. 2017;34: 148–155. doi:10.1016/j.sleep.2017.03.021

42. Lovato N, Lack L. Circadian phase delay using the newly developed re-timer portable light device. Sleep Biol Rhythms. 2016;14: 157–164. doi:10.1007/s41105-015-0034-6

43. van der Lely S, Frey S, Garbazza C, Wirz-Justice A, Jenni OG, Steiner R, et al. Blue blocker glasses as a countermeasure for alerting effects of evening light-emitting diode screen exposure in male teenagers. J Adolesc Health. 2015;56: 113–119. doi:10.1016/j.jadohealth.2014.08.002

44. Chellappa SL, Bromundt V, Frey S, Steinemann A, Schmidt C, Schlote T, et al. Association of Intraocular Cataract Lens Replacement With Circadian Rhythms, Cognitive Function, and Sleep in Older Adults. JAMA Ophthalmol. 2019;137: 878. doi:10.1001/jamaophthalmol.2019.1406

45. Nowozin C, Wahnschaffe A, Rodenbeck A, Zeeuw J de, Hädel S, Kozakov R, et al. Applying Melanopic Lux to Measure Biological Light Effects on Melatonin Suppression and Subjective Sleepiness. CAR. 2017;14. doi:10.2174/1567205014666170523094526

46. Saletu B, Dietzel M, Lesch OM, Musalek M, Walter H, Grünberger J. Effect of Biologically Active Light and Partial Sleep Deprivation on Sleep, Awakening and Circadian Rhythms in Normals. Eur Neurol. 1986;25: 82–92. doi:10.1159/000116088

47. Drennan M, Kripke DF, Gillin JC. Bright light can delay human temperature rhythm independent of sleep. American Journal of Physiology-Regulatory, Integrative and Comparative Physiology. 1989;257: R136–R141. doi:10.1152/ajpregu.1989.257.1.R136

48. Dawson D, Campbell SS. Timed Exposure to Bright Light Improves Sleep and Alertness during Simulated Night Shifts. Sleep. 1991;14: 511–516. doi:10.1093/sleep/14.6.511

49. Cajochen C, Dijk DJ, Borbély AA. Dynamics of EEG Slow-Wave Activity and Core Body Temperature in Human Sleep After Exposure to Bright Light. Sleep. 1992 [cited 8 Jun 2023]. doi:10.1093/sleep/15.4.337

50. Dumont M, Carrier J. Daytime Sleep Propensity After Moderate Circadian Phase Shifts Induced With Bright Light Exposure. Sleep. 1997;20: 11–17. doi:10.1093/sleep/20.1.11

51. Cajochen C, Kräuchi K, Danilenko KV, Wirz-Justice A. Evening administration of melatonin and bright light: Interactions on the EEG during sleep and wakefulness. Journal of Sleep Research. 1998;7: 145–157. doi:10.1046/j.1365-2869.1998.00106.x

52. Gordijn MCM, Beersma DGM, Korte HJ, Hoofdakker RH. Effects of light exposure and sleep displacement on dim light melatonin onset. J Sleep Res. 1999;8: 163–174. doi:10.1046/j.1365-2869.1999.00156.x

53. Komada Y, Tanaka H, Yamamoto Y, Shirakawa S, Yamazaki K. Effects of bright light pre-exposure on sleep onset process. Psychiatry and Clinical Neurosciences. 2000;54: 365–366. doi:10.1046/j.1440-1819.2000.00717.x

54. Burgess HJ, Sletten T, Savic N, Gilbert SS, Dawson D. Effects of bright light and melatonin on sleep propensity, temperature, and cardiac activity at night. Journal of Applied Physiology. 2001;91: 1214–1222. doi:10.1152/jappl.2001.91.3.1214

55. Kozaki T, Kitamura S, Higashihara Y, Ishibashi K, Noguchi H, Yasukouchi A. Effect of color temperature of light sources on slow-wave sleep. J Physiol Anthropol Appl Human Sci. 2005;24: 183–186. doi:10.2114/jpa.24.183

56. Münch M, Kobialka S, Steiner R, Oelhafen P, Wirz-Justice A, Cajochen C. Wavelength-dependent effects of evening light exposure on sleep architecture and sleep EEG power density in men. American Journal of Physiology-Regulatory, Integrative and Comparative Physiology. 2006;290: R1421–R1428. doi:10.1152/ajpregu.00478.2005

57. Cajochen C, Biase RD, Imai M. Interhemispheric EEG asymmetries during unilateral bright-light exposure and subsequent sleep in humans. American Journal of Physiology-Regulatory, Integrative and Comparative Physiology. 2008;294: R1053–R1060. doi:10.1152/ajpregu.00747.2007

58. Münch M, Scheuermaier KD, Zhang R, Dunne SP, Guzik AM, Silva EJ, et al. Effects on subjective and objective alertness and sleep in response to evening light exposure in older subjects. Behavioural Brain Research. 2011;224: 272–278. doi:10.1016/j.bbr.2011.05.029

59. Chellappa SL, Steiner R, Oelhafen P, Lang D, Götz T, Krebs J, et al. Acute exposure to evening blue-enriched light impacts on human sleep. J Sleep Res. 2013;22: 573–580. doi:10.1111/jsr.12050

60. Hilditch CJ, Wong LR, Bathurst NG, Feick NH, Pradhan S, Santamaria A, et al. Rise and shine: The use of polychromatic short-wavelength-enriched light to mitigate sleep inertia at night following awakening from slow-wave sleep. Journal of Sleep Research. 2022;31. doi:10.1111/jsr.13558

61. Vethe D, Drews HJ, Scott J, Engstrøm M, Heglum HSA, Grønli J, et al. Evening light environments can be designed to consolidate and increase the duration of REM-sleep. Sci Rep. 2022;12: 8719. doi:10.1038/s41598-022-12408-w

62. Spitschan M, Mead J, Roos C, Lowis C, Griffiths B, Mucur P, et al. luox: validated reference open-access and open-source web platform for calculating and sharing physiologically relevant quantities for light and lighting. Wellcome Open Res. 2022;6: 69. doi:10.12688/wellcomeopenres.16595.3
